# Investigation of Surface Properties and Antibacterial Activity of 3D-Printed Polyamide 12-Based Samples Coated by a Plasma SiO_x_C_y_H_z_ Amorphous Thin Film Approved for Food Contact

**DOI:** 10.3390/polym17121678

**Published:** 2025-06-17

**Authors:** Mario Nicotra, Raphael Palucci Rosa, Valentina Trovato, Giuseppe Rosace, Roberto Canton, Anna Rita Loschi, Stefano Rea, Mahmoud Alagawany, Carla Sabia, Alessandro Di Cerbo

**Affiliations:** 1 School of Biosciences and Veterinary Medicine, University of Camerino, 62024 Matelica, MC, Italy; mario.nicotra@unicam.it (M.N.); annarita.loschi@unicam.it (A.R.L.); stefano.rea@unicam.it (S.R.); 2 Department of Engineering and Applied Sciences, University of Bergamo, 24044 Dalmine, BG, Italy; raphael.rosa@unibg.it (R.P.R.); giuseppe.rosace@unibg.it (G.R.); 3 Local CSGI (Inter-University Center for Colloid and Surface Science) Research Unit, 24044 Dalmine, BG, Italy; 4 Local INSTM (National Consortium of Materials Science and Technology) Research Unit, 24044 Dalmine, BG, Italy; 5 Moma Nanotech Srl, 24041 Brembate, BG, Italy; canton@nanotech.it; 6 Poultry Department, Agriculture Faculty, Zagazig University, Zagazig 44511, Egypt; mmalagwany@zu.edu.eg; 7 Department of Life Sciences, University of Modena and Reggio Emilia, 41125 Modena, MO, Italy; carla.sabia@unimore.it

**Keywords:** food contact materials, additive manufacturing, food safety, SiO_x_C_y_H_z_ coating, antibacterial activity

## Abstract

Microbial contamination and biofilm formation on food contact materials (FCMs) represent critical challenges within the food supply chain, compromising food safety and quality while increasing the risk of foodborne illnesses. Traditional materials often lack sufficient microbial resistance to contamination, creating a high demand for innovative antimicrobial surfaces. This study assessed the effectiveness of a nanosized deposited SiO_x_C_y_H_z_ coating approved for food contact on 3D-printed polyamide 12 (PA12) disk substrates, aiming at providing antimicrobial and anti-biofilm functionality to mechanical components and packaging material in the food supply chain. The coating was applied using plasma-enhanced chemical vapor deposition (PECVD) and characterized through Fourier-transform infrared spectroscopy (FTIR), Raman spectroscopy, thermogravimetric analysis (TGA), scanning electron microscopy (SEM), and contact angle measurements. Coated PA12 samples exhibited significantly enhanced hydrophobicity, with an average water contact angle of 112.9°, thus improving antibacterial performance by markedly reducing bacterial adhesion. Microbiological assays revealed a significant (*p* < 0.001) bactericidal activity (up to 4 logarithms after 4 h, ≥99.99%) against Gram-positive and Gram-negative bacteria, including notable foodborne pathogens such as *L. monocytogenes*, *S. aureus, E. coli*, and *S. typhimurium*. SiO_x_C_y_H_z_-coated PA12 surfaces exhibited strong antibacterial activity, representing a promising approach for coating additive-manufactured components and equipment for packaging production in the food and pharmaceutical supply chain able to enhance safety, extend product shelf life, and reduce reliance on chemical sanitizers.

## 1. Introduction

The food supply chain (FSC), which encompasses all processes from food production to retail, continually faces considerable challenges [1]. Currently, increasing sustainability, one of the Sustainable Development Goals (SDGs) proposed by the United Nations (UN) [2], and reducing microbial contamination and the possible migration of molecules from food contact materials (FCMs) and equipment [3,4,5,6,7] are among the main challenges the FSC has to address.

Specifically, microbial contamination is one of the leading causes of food spoilage and a reduction in food quality, particularly in meat. It poses a threat to human health due to the extensive use of antibiotics in farm animals, contributing to the spread of antibiotic resistance (AR) and making animal farms a reservoir for resistant germs and genes [8].

Contamination may occur at any step of the FSC, such as fecal contamination in slaughterhouses, the use of contaminated equipment in food [9], or the use of contaminated water or sewage discharges in plant-based products [10].

Another concern for the food industry is the production of biofilms, particularly on food contact materials (FCMs). It deals with a complex microbial ecosystem where one or more microbial species, mainly bacteria and fungi, are enveloped by an extracellular matrix (made of polysaccharides, proteins, and nucleic acids) that allows them to stick to surfaces (e.g., equipment or food), creating a protective environment for the microorganisms inside [11]. Therefore, preventing biofilm formation becomes a major challenge for the food industry, where the spoilage of food and the spread of pathogens such as *Bacillus cereus*, *Campylobacter jejuni*, enterohemorrhagic *Escherichia coli*, *Listeria monocytogenes*, *Salmonella enterica*, *Staphylococcus aureus*, and *Pseudomonas* spp. represent a serious risk to consumers’ health [12,13]. For instance, *Salmonella enterica* can cause massive outbreaks with severe gastroenteric symptoms [12], while *S. aureus* can produce toxins during its growth, causing food poisoning [14], with a more severe impact on vulnerable individuals, such as children and the elderly, becoming potentially lethal. Thus, reducing food cross-contamination and the transmission of foodborne pathogens is of utmost importance, also to prevent severe economic damage that may occur due to product losses, recalls, and/or withdrawals [15].

Depending on the type of food contact surface, different cleaning methods, physical (such as brushing and scraping) and chemical (such as detergents), can be applied singly or in combination to remove dirt and germs [3].

On the other hand, to achieve effective disinfection, which involves inactivating microorganisms on surfaces, disinfectants such as iodine, biguanides, quaternary ammonium compounds, peracetic acid, sodium hydroxide, and sodium hypochlorite should be used [16]. Unfortunately, these substances often corrode the materials, compromising the integrity of the food contact surface, increasing organic matter retention, creating new binding sites for microorganisms, and making cleaning and sanitizing more challenging [17,18,19]. For this reason, ultra-high-molecular-weight polyethylene, polytetrafluoroethylene, polycarbonate, and high-density polyethylene, which are endowed with high chemical resistance, low moisture absorption, and a low coefficient of friction, are now extensively used in the food and biomedical industries [20,21]. However, due to the lack of a protective coating that can reduce the release of chemical compounds from these plastic materials, such as bisphenol A, bisphenol A diglycidyl ether, perfluorinated compounds, phthalates, primary aromatic amines, and oligomers [22], their use still poses a serious health concern [23].

To overcome the aforementioned drawbacks of both plastic- and stainless-steel-based materials, we developed a functional, nanosized SiO_x_C_y_H_z_-based coating that exhibited excellent antibacterial properties and prevented the leakage of chemical compounds from both stainless-steel and aluminum surfaces [4,5,6,7], resulting in compliance with National Sanitation Foundation/American National Standards Institute 51 (NSF/ANSI 51) [24] and Materials and Objects in Contact with Food (MOCF) [25] standards.

For this reason, our research has focused on the deposition and antibacterial activity assessment of the aforementioned SiO_x_C_y_H_z_ coating even on plastic materials such as 3D-printed polyamide 12 (PA12), a thermoplastic, medical-grade [26] (but not food-grade [27,28]) polymer with a thermostability of 185 °C, with potential use in the food chain to enhance safety, extend product shelf life, and reduce reliance on chemical sanitizers.

## 2. Materials and Methods

### 2.1. Sample Preparation

**Figure 1:**
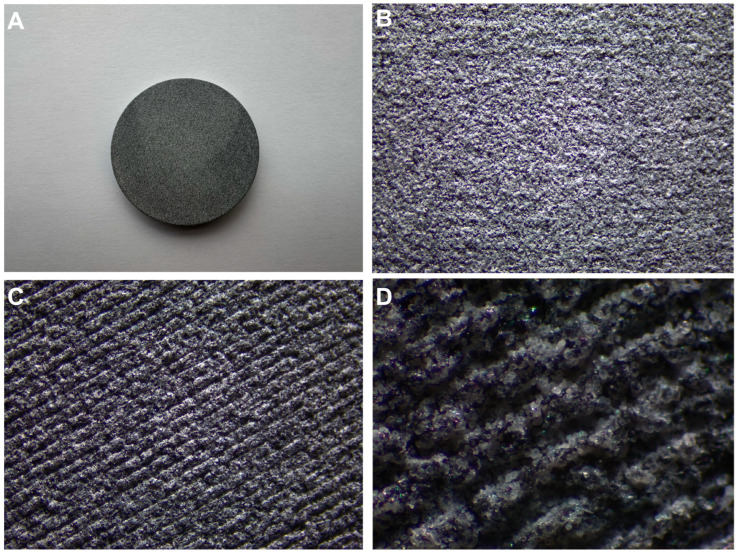
Representative (**A**) macroscopic image of PA12 disk and at different magnifications, (**B**) 8×, (**C**) 25×, and (**D**) 35×, acquired with the Leica EZ4W (Leica Microsystems Srl, Buccinasco, MI, Italy).

Eighteen round-shaped PA12 disks (Figure 1), compliant with EN ISO 10993-1:2018 [29], United States Pharmacopoeia (USP) Class VI [30], Commission Regulation (EU) 10/2011 [31], Regulation (EC) 1935/2004 [25], and Commission Regulation (EC) 2023/2006 [32], with a 4 cm diameter and a 0.5 mm thickness, were produced using a 3D printer (HP Multi Jet Fusion 5200, Manufat Srl, Lecco, Italy). The printer was supplied with HP 3D High Reusability PA12 powder (HP Inc., Palo Alto, CA, USA), described by the manufacturer as a high-reusability material optimized for additive manufacturing applications, but currently, this material cannot be used as an FCM.

**Figure 2:**
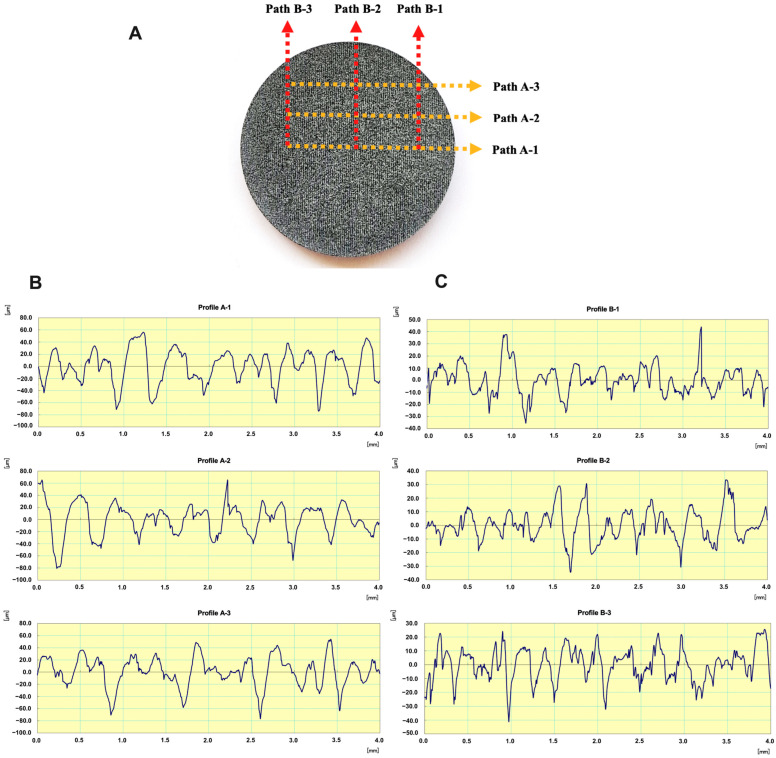
Graphical representation of (**A**) a PA12 disk with the paths of roughness, (**B**) transversal, and (**C**) longitudinal large-scale roughness measurements.

The large-scale roughness of PA12 disks was analyzed with a profilometer (SURFTEST SJ-210, Mitutoyo Italiana S.r.l., Milano, Italy) by performing an average of 3 longitudinal (20.83 ± 2.09 μm, mean ± SD) and transversal (9.03 ± 0.32 μm, mean ± SD) measurements (Figure 2).

Nine disks were left uncoated, while nine were coated with a hydrophobic, amorphous SiO_x_C_y_H_z_ transparent film (coded nanoXHAM D), suitable for food contact according to NSF/ANSI 51-2023 (USA) [24] and MOCA (CE) [25] and non-cytotoxic according to ISO 10993-5:2009 [33].

The film was deposited on the samples via plasma-enhanced chemical vapor deposition (PECVD) [34] using an industrial process chamber coupled to a 13.56 MHz radio frequency source. The coating was deposited using organosilicon and O_2_ gases at near-room temperature, establishing a stable precursor flow at approximately 0.1 kPa, and applying a radio frequency power of 300 W.

The resulting coating exhibited a thickness of about 1 µm and a surface tension of 28 mN/m according to ISO 8296-2003 [35].

### 2.2. Fourier-Transform Infrared (FTIR) Spectroscopy Analysis

Coated and uncoated PA 12 disks were characterized using FTIR spectroscopy with a Nicolet iS50 FTIR spectrometer (Thermo Fisher Scientific, Waltham, MA, USA) equipped with a diamond crystal as an internal reflectance element. The infrared analysis of coated and uncoated samples was performed by collecting spectra at room temperature in the 4000–600 cm^−1^ spectral range after 32 scans and with a resolution of 4 cm^−1^. The area of the sample and the Attenuated Total Reflection (ATR) module’s tip were properly cleansed with ethanol before setting each sample under the ATR press. Five spectra were recorded for each PA12 disk to ensure homogeneous results, and the average of the data was used for data interpretation.

### 2.3. Raman Spectroscopy Analysis

Raman spectroscopy was performed using an Xplora Plus Raman Microscope (HORIBA, Scientific, Kyoto, Italy). A 10× microscopic objective lens was used to collect the Raman signals; the laser wavelength was 785 nm with an output power of 100 mW. The spectral range was set from 350 to 3500 cm^−1^, and each measurement point acquisition time was 10 s, with 20 accumulations.

### 2.4. Thermogravimetric Analysis (TGA) and Derivative Thermogravimetric Analysis (DTG)

To evaluate the potential effects of the coating on PA12 thermal degradation and its overall thermal stability, TGA was performed employing a TGA550 analyzer (TA Instruments, New Castle, DE, USA). Specimens weighing approximately 10 mg, randomly collected in the outermost part of the printed disks, were heated from 40 °C to 750 °C at 10 °C/min in nitrogen and air atmospheres. At least three samples from each test specimen were analyzed in three independent experiments.

### 2.5. Contact Angle (CA) Analysis

To evaluate the surface wettability of the SiO_x_C_y_H_z_-coated PA12 disks, a steady-state water contact angle (CA) measurement was conducted using a homemade instrument equipped with a high-speed CCD camera. All measurements were conducted at room temperature and a relative humidity (RH) of 40 ± 5%. Cleaning procedures and CA measurements were performed using double-distilled water with a conductivity of <10^−6^ S·m^−1^ at 25 °C. Briefly, a drop of high-purity distilled water was placed onto the surfaces of coated and uncoated disks, and the image of the droplet was continuously recorded for 10 s. The CA values were determined using ImageJ software 1.42r (NIH, Bethesda, MD, USA) with the drop analysis package.

### 2.6. Scanning Electron Microscopy (SEM) and Energy Dispersive Spectroscopy (EDS) Analysis

SEM and EDS analyses were conducted using a Phenom ProX instrument (ThermoFisher Scientific, Waltham, Italy). A small part of each sample was placed on aluminum sample holders with a graphite adhesive, and SEM images of the surfaces of coated and uncoated PA12 disks were captured. Furthermore, the chemical composition and distribution of elements on uncoated and coated PA12 samples were analyzed with the energy-dispersive X-ray spectrometer (EDS) operating at a voltage of 10 keV.

### 2.7. The Bacterial Strains

Reference American Type Culture Collection (ATCC) and National Collection of Type Cultures (NCTC) strains of Gram-positive (*Staphylococcus aureus* ATCC 6538, *Enterococcus faecalis* ATCC 29212, *Listeria monocytogenes* NCTC 10888, and *Bacillus cereus* ATCC 14579) and Gram-negative (*Escherichia coli* ATCC 8739, *Pseudomonas aeruginosa* ATCC 27588, *S. typhimurium* ATCC 1402, and *Y. enterocolitica* ATCC 9610) bacteria were grown in Tryptic Soy Broth (TSB, Biolife Italiana S.r.l., Milano, Italy), incubated at 37 °C for 24 h, and then activated through two successive transfers.

### 2.8. Microbiological Analysis

In total, 100 μL of the overnight cultures of each bacterial strain were transferred to 10 mL TSB and incubated at 37 °C for 24 h. To evaluate the antibacterial activity, 400 μL of the inoculum, approximately 10^6^ Colony-Forming Units (CFU)/mL, were pipetted at the center of 3 coated and 3 uncoated PA12 disks, each previously placed into a Petri dish, spread on the surface using a sterilized spatula, and incubated for 12 h at 37 °C.

After incubation, sterile swabbing was carried out via friction of the surface at room temperature; then, under sterile conditions, the tip of the swab was put into a tube containing 1 mL of 0.9% saline, vortexed for one min, and serially tenfold diluted. Thereafter, 0.1 mL of each dilution was plated onto appropriate agar plates: Mannitol Salt Agar (Biolife Italiana S.r.l., Milano, Italy) for *S. aureus,* Kanamycin Esculin Azide Agar (Biolife Italiana S.r.l.) for *E. faecalis*, Palcam agar (Biolife Italiana S.r.l.) for *L. monocytogenes*, Tryptone Soy Agar (Biolife Italiana S.r.l.) for *Bacillus cereus*, and Mac Conkey Agar (Biolife Italiana S.r.l.) for *E. coli, Y. enterocolitica, P. aeruginosa,* and *Salmonella*. After a 24 h incubation at 37 °C, the colonies were counted.

### 2.9. Time-Course Assay and Sanitizing Procedures

For each bacterial strain, 9 SiO_x_C_y_H_z_-coated PA12 disks underwent two different sanitizing treatments, i.e., UV (UVC, 253 nm, *n* = 3) and alcohol 70% *v*/*v* (*n* = 3), while the remaining (n = 3) were used as controls. Sterile swabbing was carried out via surface friction at different times (0, 15′, 30′, 1, 2, and 4 h). Then, the swab tip was placed in a test tube with 1 mL of 0.9% saline and vortexed for one min. Serial tenfold dilutions of resuspensions were spread onto the appropriate agar plates for the viable cell count. The colonies were counted following incubation at 37 °C for 24 h.

### 2.10. Statistical Analysis

Data were analyzed using GraphPad Prism 9 software (GraphPad Software Inc., La Jolla, San Diego, CA, USA). All data are presented as means ± standard deviations. Differences in bacterial growth for each strain on uncoated and SiO_x_C_y_H_z_-coated disks were analyzed using a two-way analysis of variance (ANOVA) followed by Tukey’s multiple-comparisons test. A value of *p* < 0.05 was considered significant.

## 3. Results

### 3.1. FTIR Analysis

Within the low-pressure plasma, the precursor undergoes dissociation through inelastic collisions with electrons, resulting in the formation of smaller chemical species or radicals. A typical organosilicon precursor consists of a disiloxane (Si–O–Si) backbone, with methyl (–CH_3_) groups bonded to each silicon atom. Its dissociation primarily occurs via the cleavage of the Si–O–Si and Si–CH_3_ bonds due to electron impact within the plasma.

Structural modifications in the disks’ surface induced by plasma-deposited films were investigated by infrared spectroscopy through the identification of absorption bands, corresponding to the vibrational modes of functional groups present in the coated PA12 disks but absent in the uncoated ones.

**Figure 3:**
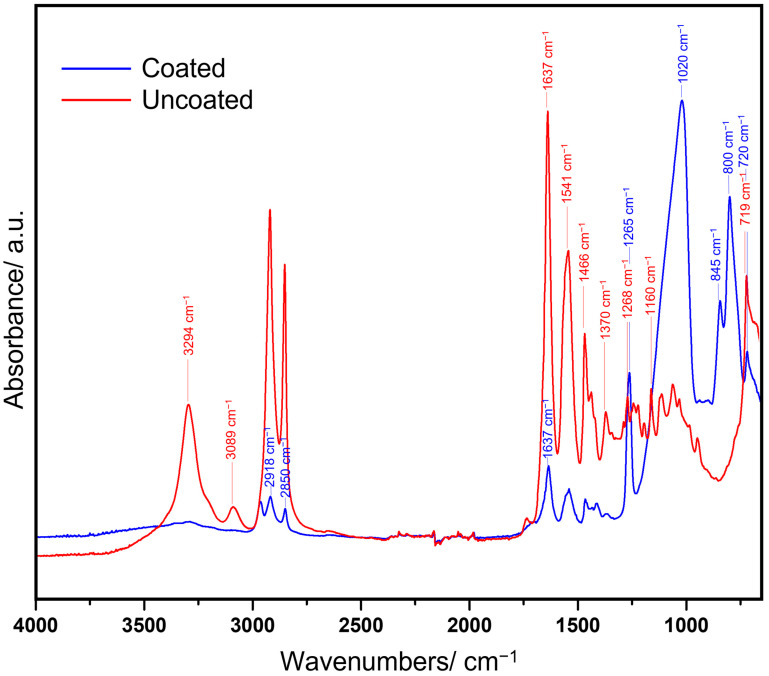
FTIR spectra of SiO_x_C_y_H_z_-coated and uncoated PA12-based disks in the range between 4000 and 530 cm^−1^.

Figure 3 shows the FTIR spectra of coated and uncoated disks in the 4000–530 cm^−1^ spectral range.

**Scheme 1:**
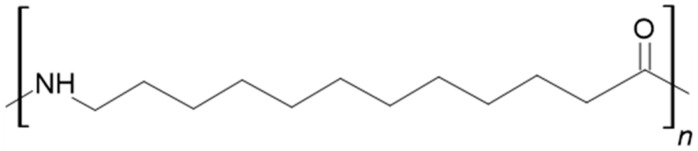
Chemical structure of PA12.

The uncoated disks exhibited an infrared spectrum typical of PA12’s chemical structure (Scheme 1), featuring characteristic absorption bands at 719, 1466, and 1637 cm^−1^, corresponding, respectively, to CH_2_ rocking, C–CO–NH_2_ bending, and C=O stretching vibrations of the primary amide. Additionally, the absorption peak at 1541 cm^−1^ can be assigned to –CN stretching coupled with C=O in-plane bending vibrations, characteristic of secondary amides, while the band at 3089 cm^−1^ is associated with the Fermi resonance of –NH_2_ stretching. Another –NH_2_ stretching vibration, indicative of primary amides, is observable at 3294 cm^−1^. Further relevant peaks at 1370, 1268, and 1160 cm^−1^ are related to CH_2_ bending and twisting, C–N stretching combined with C=O in-plane bending, and the skeletal vibrations of the CONH group, respectively [36,37,38].

Following the coating procedure, notable alterations in the spectra were observed. The coated disks exhibited a marked decrease in the intensity of bands within the ranges of 2918–2850 cm^−1^ and 1637–1369 cm^−1^, accompanied by a significant increase in the peak intensity at 1263 and 1020 cm^−1^. These enhanced peaks can be ascribed to the presence of δ (Si–(CH_3_)_x) and ν (Si–O) functional groups, respectively, indicating the successful deposition of the SiO_x_C_y_H_z_ coating [6]. Additionally, new absorption peaks emerged at 845, 800, and 720 cm^−1^, corresponding to γ (C–H), δ (Si–O), and –(CH_2_)_n rocking vibrations, respectively [6,39].

### 3.2. TGA Analysis

**Figure 4:**
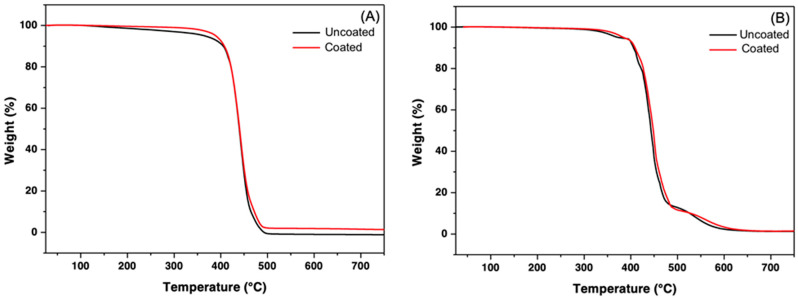
Thermogravimetric analysis of uncoated and SiO_x_C_y_H_z_-coated disks under nitrogen (**A**) and air (**B**) atmospheres.

The thermal stability of a polymer determines its effective operational temperature range and can be accurately assessed using thermogravimetric analysis (TGA) in a nitrogen atmosphere. As illustrated in Figure 4A, this process exhibited a single-step decomposition pattern, with the uncoated PA12 maintaining thermal stability up to approximately 350 °C and thermal degradation occurring between 400 and 500 °C, producing negligible residue weight, indicating that pure PA shows good stability but is predominantly pyrolyzed at high temperatures. The coated PA12 disks demonstrated improved thermal stability, with the onset of decomposition shifted to around 430 °C and a slightly higher final residue of approximately 2–3%. The increased thermal stability of coated PA12 may be attributed to the hydrogen bonds between the amide group of PA and the hydroxyl group of the SiO_x_C_y_H_z_ coating, which strengthens the interfacial interaction and requires more energy to break the bonds. However, the presence of the coating did not significantly affect the degradation mechanism.

In an oxidative atmosphere, the thermal degradation of polyamides occurs through a two-step process: the initial depolymerization at the chain end groups, followed by chain scission, which leads to the formation of unsaturated nitriles and alkenes. The latter step is significantly accelerated in the presence of oxygen [40].

Accordingly, the investigated uncoated PA12 disks (Figure 4B) underwent this two-step decomposition process, with the onset of decomposition at approximately 350 °C.

This was followed by a substantial, distinct secondary degradation event between 400 °C and 500 °C, resulting in a negligible final residue of about 0–1%. A secondary peak appeared at approximately 560 °C, suggesting an additional thermal event corresponding to the oxidation of carbonaceous residues formed during the primary decomposition process or potentially arising from additives, stabilizers, or impurities within the disks.

While demonstrating moderately improved stability with an onset at 370 °C, the coated disks still exhibited a residue of less than 1%.

Similar to the findings observed in a nitrogen atmosphere, the results suggest that the presence of the PECVD-assisted amorphous coating did not significantly alter the degradation mechanism of PA12 in air. The thin film did not provide adequate thermal shielding to mitigate heat transfer and did not notably slow the diffusion of volatile degradation products, consequently allowing for the carbonization of the polymer. As such, the deposition of the amorphous coating did not substantially influence the thermal stability or resistance to thermo-oxidative degradation of the coated disks.

### 3.3. Raman Characterization

**Figure 5:**
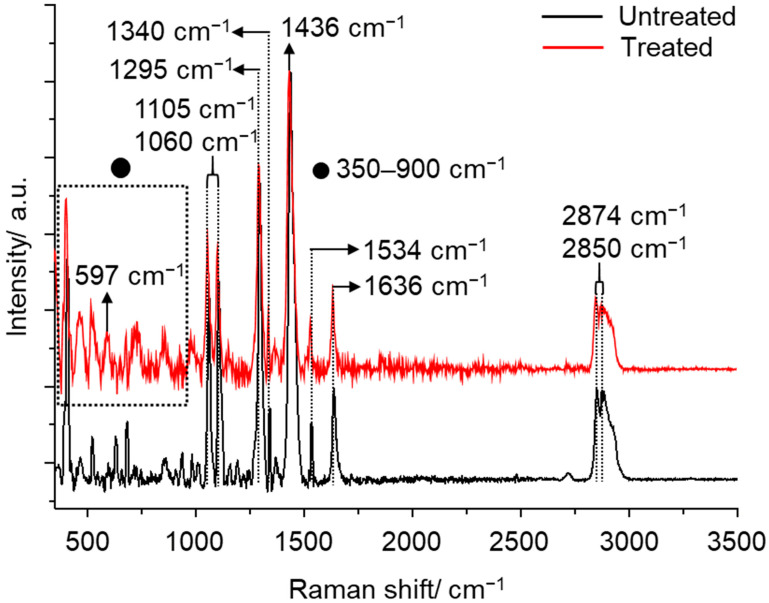
Raman spectra of uncoated and SiO_x_C_y_H_z_-coated disks in the spectral range 350–3500 cm^−1^. The dashed box, marked with a black dot, highlights the 350–900 cm^−1^ range.

To ascertain whether the plasma-induced coating changed the surface polymer structure, uncoated and coated PA12 disks were compared using Raman spectroscopy (Figure 5). In the high wavenumber region of the uncoated disks, bands were observed between 2875 and 2850 cm^−1^, corresponding to the symmetric and asymmetric stretching vibrations of methylene (CH_2_) groups. In the fingerprint region (approximately 500–1700 cm^−1^), numerous distinct vibrational modes can be identified. The band at 1636 cm^−1^ corresponds to the amide I peak (C=O stretching vibration), while the peak observed around 1295 cm^−1^ originates from the amide III vibration, primarily involving C-N stretching coupled with N-H bending [41]. In the medium wavenumber region, bands at 1436, 1340, 1105, and 1060 cm^−1^ were ascribed to the scissoring and bending vibrations of CH_2_ groups within the alkyl chains, as well as the stretching vibrations of C-O-C bonds, respectively [42,43]. In the coated PA12 disks, new bands appeared in the range between 350 and 900 cm^−1^, ascribed to Si–O–Si mixed symmetric stretching and bending vibrations (300–500 cm^−1^) and Si–O–Si rocking vibrations (597 cm^−1^) [44,45], indicating the presence of the SiO_x_C_y_H_z_ coating on the surface.

### 3.4. CA Analysis

**Figure 6:**
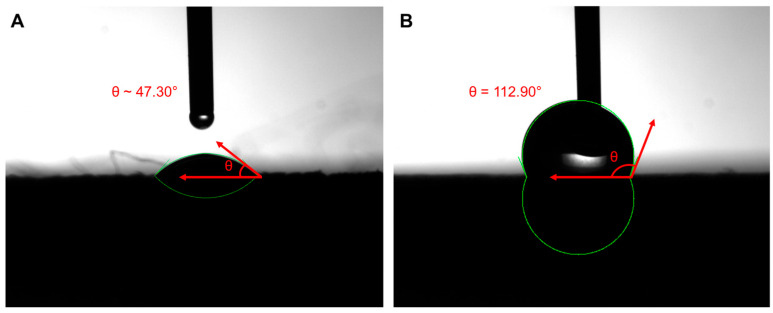
Steady-state water contact angle measurement of (**A**) uncoated and (**B**) SiO_x_C_y_H_z_-coated disks. The green lines indicate the areas used for contact angle measurements, which were highlighted in red.

The surface wettability was investigated through steady-state water contact angle (CA) measurements, with images of the uncoated and coated disks presented in Figure 6A and B, respectively.

The SiO_x_C_y_H_z_-coated disks exhibited lower wettability than the uncoated ones, as evidenced by a mean CA value of 112.90° ± 1.75 (Table 1). Conversely, the uncoated disks showed a mean CA value of less than 90° (47.30 ± 0.67), probably due to the high porosity of the material and its hydrophilicity, which prevented any further analysis due to the immediate absorption of the drop.

### 3.5. SEM and EDS Analysis

**Figure 7:**
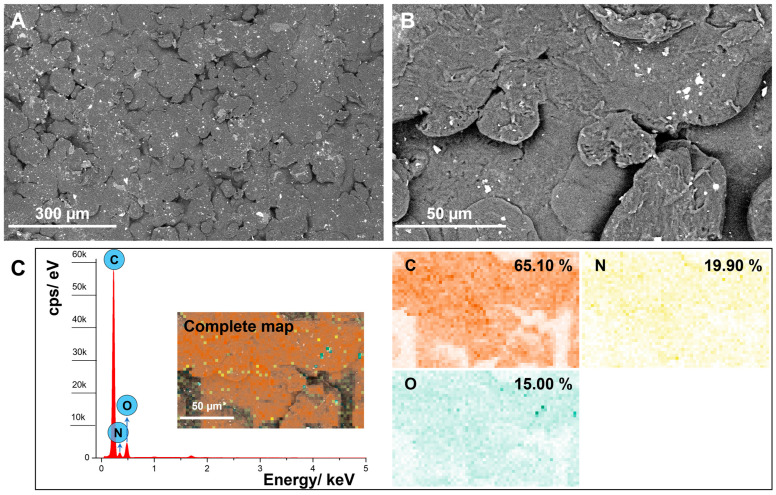
SEM images at different magnifications (**A**,**B**) and EDS elemental maps along with corresponding quantitative analysis (reported in weight percent, wt%) (**C**) of uncoated PA12 disks.

**Figure 8:**
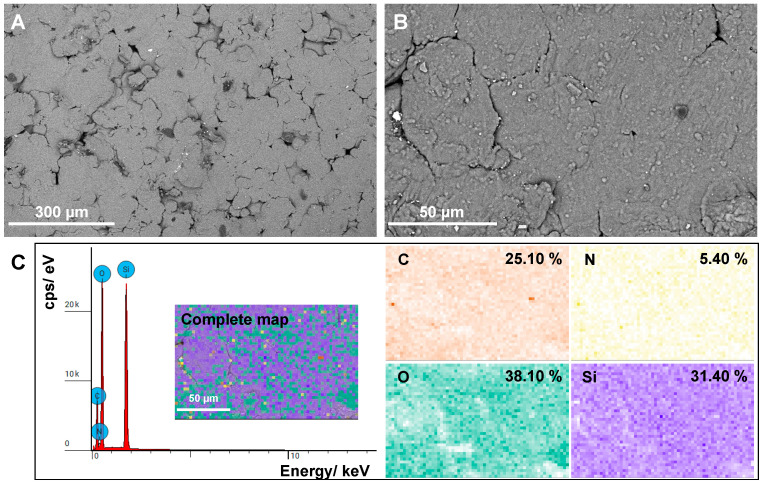
SEM images at different magnifications (**A**,**B**) and EDS elemental maps along with corresponding quantitative analysis (reported in weight percent, wt%) (**C**) of SiO_x_C_y_H_z_-coated PA12 disk.

SEM and EDS analyses of the uncoated and coated disks are shown in Figure 7 and Figure 8, respectively. Figure 7A shows the surface morphology of the uncoated disk, which appears uniform but porous, as further confirmed by the higher-magnification images (Figure 7B). The chemical composition, obtained by EDS microanalysis (Figure 7C), is illustrated through elemental maps showing the relative distribution of the detected elements: orange, yellow, and green correspond to the presence of carbon, nitrogen, and oxygen, respectively. The quantitative data derived from EDS analysis (Figure 7C) indicate that carbon is the predominant element in the uncoated PA12 disks, followed by nitrogen and oxygen.

Conversely, the surface morphology of the coated disks shown in Figure 8A looks more homogeneous and less porous compared to the uncoated sample, as further evidenced by the higher-magnification image that reveals a smoother and more compact surface structure (Figure 8B). Quantitative EDS analysis (Figure 8C) shows that the coated disks’ composition differs from that of the uncoated disks, with silicon emerging as a major component, as well as oxygen. These results indicate the successful deposition of the silicon-based coating, which significantly modifies both the chemical and morphological characteristics of the PA12 surface.

### 3.6. Microbiological Analysis

In Figure 8, differences in bacterial growth between uncoated and SiO_x_C_y_H_z_-coated PA12 disks challenged for 12 h with Gram-positive and Gram-negative strains are represented.

**Figure 9:**
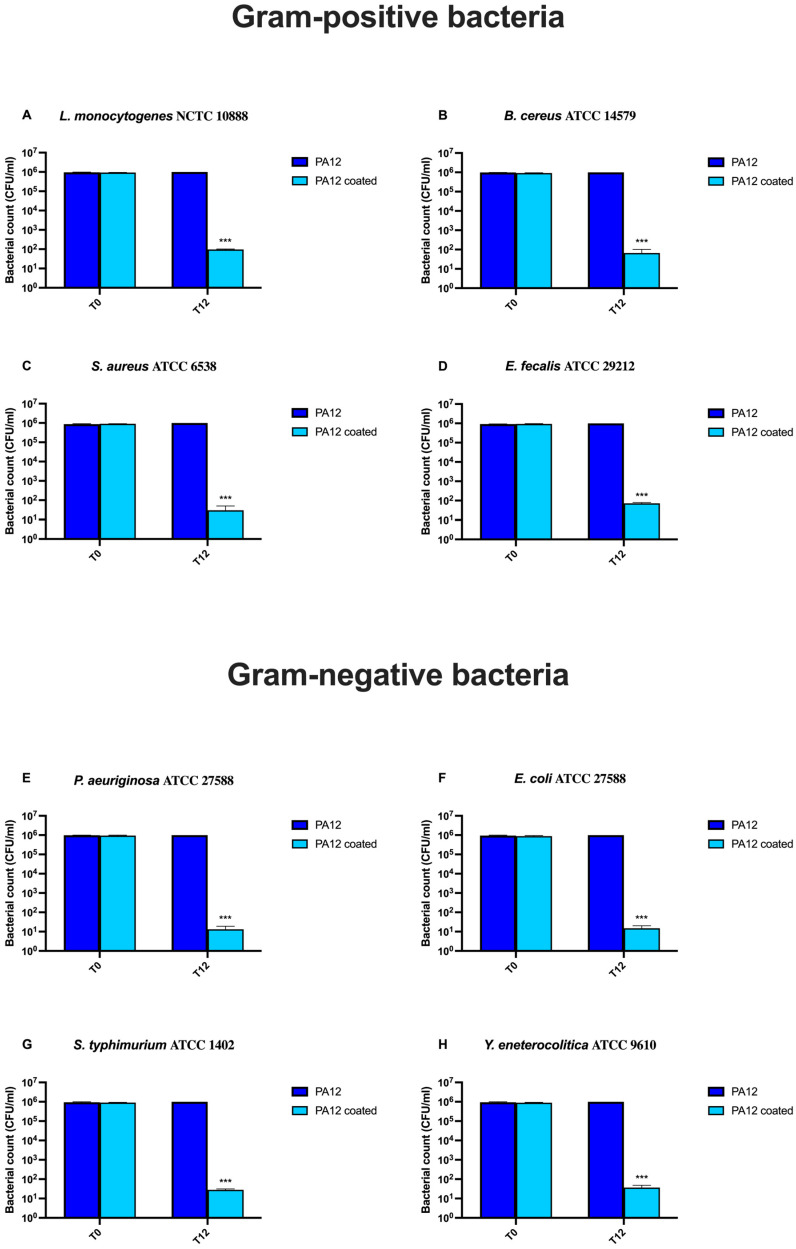
Graphical representation of the different antibacterial activities of uncoated and SiO_x_C_y_H_z_-coated PA12 disks against Gram-positive and Gram-negative bacteria—(**A**) *L. monocytogenes* NCTC 10888, (**B**) *B. cereus* ATCC 14579, (**C**) *S. aureus* ATCC 6538, (**D**) *E. faecalis* ATCC 29212, (**E**) *P. aeruginosa* ATCC 27588, (**F**) *E. coli* ATCC 8739, (**G**) *S. typhimurium* ATCC 1402, and (**H**) *Y. enterocolitica* ATCC 9610—after 12 h incubation. *** *p* < 0.001.

An overall significant decrease in Gram-positive bacteria growth was observed at 12 h for SiO_x_C_y_H_z_-coated PA12 disks with respect to the controls (*p* < 0.001, Figure 9A–D). As for *L. monocytogenes* NCTC 10888, the load decreased from 9.4 × 10^5^ CFU/mL to 9.6 × 10^1^ CFU/mL; for *B. cereus* ATCC 14579, the load decreased from 9.2 × 10^5^ CFU/mL to 6.7 × 10^1^ CFU/mL; for *S. aureus* ATCC 6538, the load decreased from 9.03 × 10^5^ CFU/mL to 3.0 × 10^1^ CFU/mL; and for *E. faecalis* ATCC 29212, the load decreased from 9.4 × 10^5^ CFU/mL to 7.2 × 10^1^ CFU/mL.

As observed for Gram-positive bacteria, a significant decrease in bacterial growth was observed after 12 h for SiO_x_C_y_H_z_-coated PA12 disks compared to the controls (*p* < 0.001, Figure 9E–H). As for *P. aeruginosa* ATCC 27588, the load decreased from 9.46 × 10^5^ CFU/mL to 1.33 × 10^1^ CFU/mL; for *E. coli* ATCC 8739, the load decreased from 8.8 × 10^5^ CFU/mL to 1.5 × 10^1^ CFU/mL; for *S. typhimurium* ATCC 1402, the load decreased from 9.13 × 10^5^ CFU/mL to 2.83 × 10^1^ CFU/mL; and for *Y. enterocolitica* ATCC 9610, the load decreased from 9.0 × 10^5^ CFU/mL to 3.66 × 10^1^ CFU/mL.

### 3.7. Time-Course Assay

**Figure 10:**
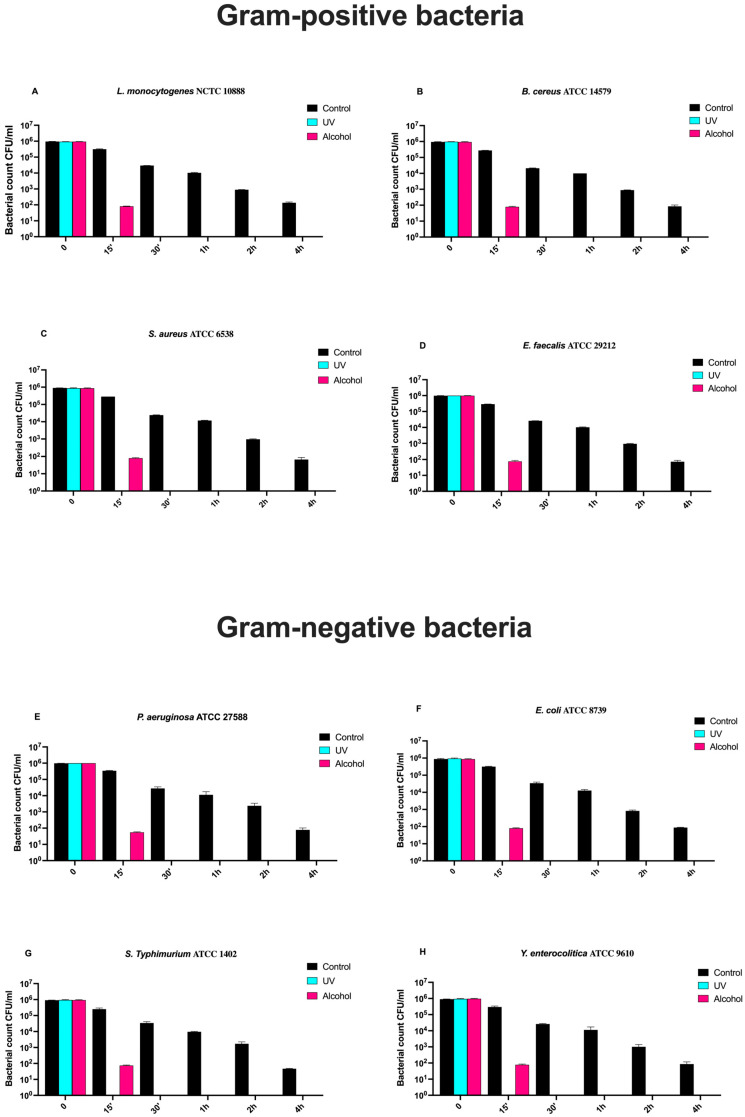
Graphical representation of the different exposure times of SiO_x_C_y_H_z_-coated PA12 disks unsanitized (control) and subjected to two sanitizing methods (UVC and alcohol) against (**A**) *Listeria monocytogenes* NCTC 10888, (**B**) *Bacillus cereus* ATCC 14579, (**C**) *Staphylococcus aureus* ATCC 6538, (**D**) *Enterococcus faecalis* ATCC 29212, (**E**) *P. aeruginosa* ATCC 27588, (**F**) *E. coli* ATCC 8739, (**G**) *S. typhimurium* ATCC 1402, and (**H**) *Y. enterocolitica* ATCC 9610 after 12 h incubation.

Figure 10 reports the Gram-positive and Gram-negative loads (ordinates) on SiO_x_C_y_H_z_-coated PA12 disks as a function of the different exposure times (abscissa). Black, turquoise, and purple bars refer to control (untreated with disinfectant), UVC-, and alcohol-treated disks, respectively.

Regardless of the bacterial strain, all SiO_x_C_y_H_z_-coated PA12 disks used as controls induced a four-logarithm decrease from 10^6^ to 10^2^ after 4 h, UVC eliminated any bacterial load already after 15 min, and alcohol achieved a similar result after 30 min.

As for *L. monocytogenes* NCTC 10888, the bacterial load present on the SiO_x_C_y_H_z_-coated PA12 control disks decreased from 9.53 × 10^5^ CFU/mL at T0 to 3.16 × 10^5^ CFU/mL after 15 min, to 2.9 × 10^4^ CFU/mL after 30 min, to 1.03 × 10^3^ CFU/mL after 1 h, to 8.93 × 10^2^ CFU/mL after 2 h, and to 1.36 × 10^2^ CFU/mL after 4 h (Figure 10A). Conversely, the bacterial load on the SiO_x_C_y_H_z_-coated PA12 disks sanitized with alcohol decreased from 9.33 × 10^5^ CFU/mL at T0 to 8.16 × 10^1^ CFU/mL after 15 min. Dealing with *B. cereus* ATCC 14579, the load decreased from 9.5 × 10^5^ at T0 to 2.76 × 10^5^ CFU/mL after 15 min, to 2.1 × 10^4^ CFU/mL after 30 min, to 1.0 × 10^4^ CFU/mL after 1 h, to 8.83 × 10^2^ CFU/mL after 2 h, and to 8.5 × 10^1^ CFU/mL after 4 h (Figure 10B). As previously reported, the bacterial load on the SiO_x_C_y_H_z_-coated PA12 disks sanitized with alcohol decreased from 9.33 × 10^5^ CFU/mL at T0 to 8.0 × 10^1^ CFU/mL after 15 min. Considering *Staphylococcus aureus* ATCC 6538, the load decreased from 9.03 × 10^5^ at T0 to 2.85 × 10^5^ CFU/mL after 15 min, to 2.43 × 10^4^ CFU/mL after 30 min, to 1.16 × 10^4^ CFU/mL after 1 h, to 9.66 × 10^2^ CFU/mL after 2 h, and to 6.67 × 10^1^ CFU/mL after 4 h (Figure 10C). As reported for the other two previous strains, the bacterial load on the SiO_x_C_y_H_z_-coated PA12 disks sanitized with alcohol decreased from 8.73 × 10^5^ CFU/mL at T0 to 8.16 × 10^1^ CFU/mL after 15 min. As for *Enterococcus faecalis* ATCC 29212, the load decreased from 9.83 × 10^5^ at T0 to 2.91 × 10^5^ CFU/mL after 15 min, to 2.63 × 10^4^ CFU/mL after 30 min, to 1.03 × 10^4^ CFU/mL after 1 h, to 9.33 × 10^2^ CFU/mL after 2 h, and to 7.33 × 10^1^ CFU/mL after 4 h (Figure 10D). The bacterial load on the SiO_x_C_y_H_z_-coated PA12 disks sanitized with alcohol decreased from 9.90 × 10^5^ CFU/mL at T0 to 7.66 × 10^1^ CFU/mL after 15 min.

Dealing with *P. aeruginosa* ATCC 27588, the load decreased from 9.93 × 10^5^ at T0 to 3.4 × 10^5^ CFU/mL after 15 min, to 2.8 × 10^4^ CFU/mL after 30 min, to 1.13 × 10^4^ CFU/mL after 1 h, to 2.38 × 10^2^ CFU/mL after 2 h, and to 7.83 × 10^1^ CFU/mL after 4 h (Figure 10E). The bacterial load on the SiO_x_C_y_H_z_-coated PA12 disks sanitized with alcohol decreased from 1 × 10^6^ CFU/mL at T0 to 5.5 × 10^1^ CFU/mL after 15 min. Considering the *E. coli* ATCC 8739, the load decreased from 8.8 × 10^5^ at T0 to 3.16 × 10^5^ CFU/mL after 15 min, to 3.41 × 10^4^ CFU/mL after 30 min, to 1.26 × 10^4^ CFU/mL after 1 h, to 8.27 × 10^2^ CFU/mL after 2 h, and to 9.83 × 10^1^ CFU/mL after 4 h (Figure 10F).

The bacterial load on the SiO_x_C_y_H_z_-coated PA12 disks sanitized with alcohol decreased from 8.66 × 10^5^ CFU/mL at T0 to 8.0 × 10^1^ CFU/mL after 15 min. As for *S. typhimurium* ATCC 1402, the load decreased from 9.13 × 10^5^ at T0 to 2.53 × 10^5^ CFU/mL after 15 min, to 3.43 × 10^4^ CFU/mL after 30 min, to 9.66 × 10^3^ CFU/mL after 1 h, to 1.7 × 10^3^ CFU/mL after 2 h, and to 4.67 × 10^1^ CFU/mL after 4 h (Figure 10G). The bacterial load on the SiO_x_C_y_H_z_-coated PA12 disks sanitized with alcohol decreased from 9.40 × 10^5^ CFU/mL at T0 to 7.5 × 10^1^ CFU/mL after 15 min. As for *Y. enterocolitica* ATCC 9610, the load decreased from 9.0 × 10^5^ at T0 to 2.96 × 10^5^ CFU/mL after 15 min, to 2.63 × 10^4^ CFU/mL after 30 min, to 1.13 × 10^4^ CFU/mL after 1 h, to 1.02 × 10^3^ CFU/mL after 2 h, and to 8.66 × 10^1^ CFU/mL after 4 h (Figure 10H). The bacterial load on the SiO_x_C_y_H_z_-coated PA12 disks sanitized with alcohol decreased from 9.6 × 10^5^ CFU/mL to 7.83 × 10^1^ CFU/mL after 15 min.

## 4. Discussion

The current study assessed the potential antibacterial effect of a SiO_x_C_y_H_z_-based coating (coded nanoXHAM-D) applied to PA12 disks by low-pressure plasma, with or without the combined application of two sanitizing treatments (UVC and alcohol 70% *v*/*v*).

All coated disks that did not undergo sanitization showed a statistically significant decrease in bacterial load after 12 h, although the time course showed that such a result was already achieved in 4 h. Conversely, no bacterial reduction occurred in the same conditions on uncoated disks.

Regarding the two sanitizing treatments, UV and alcohol, the time course showed complete surface sanitization in all coated disks after 15 and 30 min, respectively.

The observed effect could be attributed to the high hydrophobicity of the nanosized coating, as confirmed by the CA angle mean value of 112.90°, which can prevent bacterial adhesion [46].

The slightly different behavior among bacterial strains can be attributed to their varying hydrophobicity. Previous research has shown a direct correlation between surface hydrophobicity and bacterial cell walls, with hydrophobic bacteria adhering to hydrophobic surfaces possibly through interactions between the substrate and bacterial surface proteins, thereby enhancing hydrophobic forces and favoring bacterial adhesion [47,48,49].

Subsequently, the variations in bacterial hydrophobicity may explain why certain colonies can still adhere more to the coated surfaces despite the significant hydrophobicity of the coating.

Besides preventing bacterial adhesion, the coating activity becomes fundamental in preventing biofilm formation, thereby reducing the need for sanitizing treatments and, consequently, the onset of treatment resistance. Biofilm formation generally occurs after bacterial adhesion on a surface, resulting from physicochemical and molecular interactions between the microorganism and the surface [50,51]. It involves a two-phase process, where the first phase consists of reversible adhesion, primarily caused by physical contact between the microorganism and the surface. The second phase is a time-dependent phase, during which molecular and cellular interactions predominate, resulting in increased adhesion [52]. Thereafter, bacteria aggregate and produce the extracellular polymeric substance, essentially made of extracellular proteins, DNA, polysaccharides, surfactants, lipids, and water, which protects the microorganisms from several environmental stresses, including antimicrobials and disinfectants, and plays a crucial role in the biofilm maturation process [53].

The present results agree with those previously achieved with the same coating deposited on stainless-steel disks with three different surface roughnesses, tested against four Gram-positive and four Gram-negative strains [4,6]. All the coated disks demonstrated a statistically significant antibacterial effect, which was further enhanced by applying the same sanitizing treatments tested in the current study. Furthermore, the bacterial load decreased by five logarithms within 6 h without disinfection, and complete sanitization was achieved 1 min and 15 s after the application of alcohol and UV, respectively. As also hypothesized in these previous studies, the high hydrophobicity and, consequently, the low surface tension of the coating could be directly responsible for its anti-adhesive activity.

Our results also partially agree with those of Fonseca et al. (2022), who demonstrated the antibacterial effect of a SiO_2_-based nano-coating on different polymeric biomaterials, such as poly(vinyl chloride) (PVC), polyurethane, and silicone, suggesting its usefulness in preventing transfusion-transmitted infections [54]. Such a nanosized coating resulted in a more than 90% reduction in bacterial concentration in nearly all tested samples, with some differences observed between Gram-positive and Gram-negative bacteria. In particular, a 2-log reduction was observed in a nutrient broth of PVC challenged with *Staphylococcus aureus* and a 5-log decrease in PVC challenged with *Escherichia coli*; a 5-log decrease in polyurethane challenged with *Staphylococcus aureus* and a 3-log decrease in polyurethane challenged with *Escherichia coli*; and a 5-log decrease in silicone challenged with *Staphylococcus aureus* or *Escherichia coli*. Conversely, our research demonstrated a homogeneous antibacterial effect against both Gram-positive and Gram-negative bacteria. Moreover, although Fonseca et al. ascribed this different behavior to an extended interaction of SiO_2_ nanoparticles with peptidoglycan saccharides on the Gram-positive bacterial surface, they ruled out the possible involvement of any compound release from the coating, as the cell viability was >90%. In this sense, based on our previous observations regarding the lack of chemical compounds released from the coating [4], this research aligns with that of Fonseca et al., confirming that the antibacterial effect of the nanosized SiO_x_C_y_H_z_ coating (coded nanoXHAM-D) is independent of the material used.

Similarly to our study, Kumar et al. (2005) exploited the hygroscopicity of the polyamide by filling the polymer with Ag^+^ ions to obtain an antibacterial material [55]. However, a significant decrease in the bacterial load against *Staphylococcus aureus* and *Escherichia coli*, 2 and 3 log, respectively, was observed only after 28 days and was mainly attributed to the higher Ag^+^ release (2.75 × 10^−4^ g/L). Ag^+^ release was also considered as one of the leading causes of the antibacterial activity of silver-doped organic–inorganic hybrid coatings prepared from tetraethoxysilane- and triethoxysilane-terminated poly (ethylene glycol)-block-polyethylene and deposited onto polyethylene (PE) and PVC [56]. More specifically, a 6-log reduction was observed for *Staphylococcus aureus* and *Escherichia coli* starting from 10^6^ CFU/mL within 6 h. Notably, the authors ascribed the antibacterial activity to the release of silver ions (3.7 × 10^−7^ g/L per square meter of coating).

More recently, a study by Piccioni et al. (2025) observed a significant antibacterial activity of polypyrrole at different concentrations (2, 4, and 6 g/L) deposited onto cotton and polyamide 6,6 fabrics and challenged with *Staphylococcus aureus* and *Escherichia coli* for 12 min, 30 min, and 1 h [57]. Starting from an inoculum concentration ranging from 1.5 and 3.0 × 10^5^ CFU/mL, the authors observed that the bacterial load reduction (>90%) was mostly influenced by the intrinsic behavior of the two materials rather than the polypyrrole concentration. Despite both materials having comparable water contact angle values when coated by polypyrrole, polyamide 6,6, which is hydrophobic, absorbed less bacterial inoculum than cotton, which is hydrophilic, thus explaining the lack of a direct correlation between the polypyrrole amount in polyamide 6,6 and bacterial reduction. On the contrary, cotton, able to soak more bacterial inoculum in a few seconds, enhanced the contact between polypyrrole and the bacteria and showed the best performance regardless of the strain.

Unlike the aforementioned works, our research widened the panel of microorganisms studied, neutralizing the impact of the material below the coating and homogenizing the effect on both Gram-positive and Gram-negative bacteria. Moreover, despite studies assessing the possible leakage of silver or zinc oxide nanoparticles embedded in plastic material, they could not prevent it, posing a potential risk to end-users’ health. In this sense, our study demonstrated the great potential of a coating that can be safely employed in the food and biomedical industries due to its ability to isolate and exhibit bactericidal activity.

## 5. Conclusions

This study demonstrates that a nanosized SiO_x_C_y_H_z_ coating approved for food contact and deposited on 3D-printed PA12 surfaces effectively conferred significant bactericidal activity with an overall 4-logarithm (≥99.99%) decrease in bacterial load.

This effect can be primarily ascribed to the increased hydrophobicity of the coating, as confirmed by contact angle and surface tension measurements, thus highlighting its intrinsic bactericidal activity. These results also confirm the potential applicability of the coating in the “additive manufacturing”, making it a promising solution for components and equipment for packaging production in the biomedical and food industries. Moreover, such a coating could enhance food safety, reduce the incidence of foodborne diseases, and minimize reliance on sanitizing treatments, closely merging with current sustainability and public health goals.

Nevertheless, further investigations into the application of such coatings on more complex surfaces of PA12-based components (e.g., joints, belt conveyors, bottling lines, food cabinets, star wheels, thrust washers, valves, and fittings) under industrial conditions are needed to validate these results.

## Figures and Tables

**Table 1:** Steady-state water drop properties.

	Contact Angle (°)	Drop Volume (mm^3^)	Drop Surface (mm^2^)	Contact Surface (mm^2^)
Sample 1	111.374	4.02	9.94	3.40
Sample 2	114.825	4.31	10.5	3.32
Sample 3	112.518	3.05	8.35	2.58
Mean ± SD	112.90 ± 1.76	3.79 ± 0.66	9.60 ± 1.11	3.10 ± 0.45

## Data Availability

The original contributions presented in this study are included in the article. Further inquiries can be directed to the corresponding authors.

## Abbreviations

The following abbreviations are used in this manuscript:

PA12	Polyamide 12
PECVD	Plasma-enhanced chemical vapor deposition
FTIR	Fourier-transform infrared spectroscopy
TGA	Thermogravimetric analysis
SEM	Scanning electron microscopy
FSC	Food supply chain
SDGs	Sustainable Development Goals
FCMs	Food contact materials
AR	Antibiotic resistance
ATR	Attenuated total reflection
CA	Contact angle
EDS	Energy dispersive spectroscopy
ATCC	American Type Culture Collection
CFU	Colony-forming units
